# Supplementary material for: Network design principle for robust oscillatory behaviors with respect to biological noise
Source: eLife. 2022 Sep 20;11:e76188. doi: 10.7554/eLife.76188 (PMC9489215; doi:10.7554/eLife.76188)
Supplement: Supplementary file 1. — (a) Parameters for searching topologies. (b) Parameters used in Figure 4. (c) Parameters used in Figure 5. [file elife-76188-supp1.docx]

**Supplementary file 1a: Parameters for searching topologies**

| $v_{ij},(i,j=1,2,3)$ | ${10}^{x}$, where $x$ is uniformly distributed in [0,2] |
| --- | --- |
| $K_{ij},(i,j=1,2,3)$ | ${10}^{x}$, where $x$ is uniformly distributed in [-2,2] |
| $\delta_{i},(i=1,2,3)$ | ${10}^{x}$, where $x$ is uniformly distributed in [-1,1] |
| $r_{i},(i=1,2,3)$ | ${10}^{x}$, where $x$ is uniformly distributed in [0,2] |
| $\tau^{noise}$ | $1$ |
| $\Delta t$ | ${10}^{-3}T$, where $T$ is the period length |
| $\sigma$ | $1$ |
| $\varepsilon$ for extrinsic noise | $0.5$ |
| $V$ for intrinsic noise | $100$ |

**Supplementary file 1b: Parameters used in Figure 4**

| $v_{BA}$ | $4.5$ |
| --- | --- |
| $v_{AB}$ | $30.5$ |
| $v_{BB}$ | $7$ |
| $K_{BA}$ | $0.42$ |
| $K_{AA}$ | $0.03$ |
| $K_{BB}$ | $0.07$ |
| $r_{A}$ | 0.4 |
| $r_{B}$ | 0.5 |
| $\delta_{A},\delta_{B}$ | $0$ |
| $\tau_{noise}$ | $1$ |
| $\sigma$ | $1$ |
| $\varepsilon$ for extrinsic noise | $0.5$ |
| $V$ for intrinsic noise | $100$ |

**Supplementary file 1c: Parameters used in Figure 5**

| Activator-inhibitor | |
| --- | --- |
| $v_{BA}$ | $18.8$ |
| $v_{AB}$ | $2.8$ |
| $v_{BB}$ | $44.8$ |
| $K_{BA}$ | $0.15$ |
| $K_{AA}$ | $0.016$ |
| $K_{BB}$ | $0.03$ |
| $r_{A}$ | $1.3$ |
| $r_{B}$ | $4.3$ |
| $\delta_{A},\delta_{B}$ | $0$ |
| $\tau_{noise}$ | $1$ |
| $\sigma$ | $1$ |
| $\varepsilon$ for extrinsic noise | $0.5$ |
| $V$ for intrinsic noise | $100$ |
| B inhibits C, and the latter inhibits A | |
| $v_{BC}$ | $3.1$ |
| $K_{BC}$ | $1.26$ |
| $K_{CA}$ | the inverse of $1,5,10,20,30,30$, and $50$ |
| $\delta_{C}$ | $10$ |
